# Cytogenetic analysis of an exposed-referent study: perchloroethylene-exposed dry cleaners compared to unexposed laundry workers

**DOI:** 10.1186/1476-069X-10-16

**Published:** 2011-03-10

**Authors:** James D Tucker, Karen J Sorensen, Avima M Ruder, Lauralynn Taylor McKernan, Christy L Forrester, Mary Ann Butler

**Affiliations:** 1 Department of Biological Sciences, 2117 Biological Sciences Building, 5047 Gullen Mall, Wayne State University, Detroit, MI 48202-3917 USA; 2 Physical and Life Sciences Directorate, L-452, Lawrence Livermore National Laboratory, Livermore, CA 94550 USA; 3 National Institute for Occupational Safety and Health, CDC, 4676 Columbia Parkway, Cincinnati, OH 45226 USA; 4 National Institute for Occupational Safety and Health, CDC, CDCW Bldg PATRI Room 9271, Washington, DC 20201 USA

## Abstract

**Background:**

Significant numbers of people are exposed to tetrachloroethylene (perchloroethylene, PCE) every year, including workers in the dry cleaning industry. Adverse health effects have been associated with PCE exposure. However, investigations of possible cumulative cytogenetic damage resulting from PCE exposure are lacking.

**Methods:**

Eighteen dry cleaning workers and 18 laundry workers (unexposed controls) provided a peripheral blood sample for cytogenetic analysis by whole chromosome painting. Pre-shift exhaled air on these same participants was collected and analyzed for PCE levels. The laundry workers were matched to the dry cleaners on race, age, and smoking status. The relationships between levels of cytological damage and exposures (including PCE levels in the shop and in workers' blood, packyears, cumulative alcohol consumption, and age) were compared with correlation coefficients and t-tests. Multiple linear regressions considered blood PCE, packyears, alcohol, and age.

**Results:**

There were no significant differences between the PCE-exposed dry cleaners and the laundry workers for chromosome translocation frequencies, but PCE levels were significantly correlated with percentage of cells with acentric fragments (R^2 ^= 0.488, p < 0.026).

**Conclusions:**

There does not appear to be a strong effect in these dry cleaning workers of PCE exposure on persistent chromosome damage as measured by translocations. However, the correlation between frequencies of acentric fragments and PCE exposure level suggests that recent exposures to PCE may induce transient genetic damage. More heavily exposed participants and a larger sample size will be needed to determine whether PCE exposure induces significant levels of persistent chromosome damage.

## Background

Tetrachloroethylene (perchloroethylene; PCE; CAS No. 127-18-4) is a chlorinated solvent with widespread use as a degreasing agent and as a solvent in the dry cleaning industry. Over 1.5 million workers are exposed to this compound every year [1], with historical mean occupational exposure levels of 59 ppm and higher depending on the application [2]. While current mean levels of exposure to PCEs are substantially lower, probably in the range of 1-10 ppm, concerns about exposures continue because PCE is an established animal carcinogen [3,4] causing leukemias and kidney and liver tumors. IARC considers PCE to be a probable human carcinogen and a definite animal carcinogen [5]. PCE is one of twenty common occupational carcinogens "where evidence of carcinogenicity is substantial but not yet conclusive for humans", and for which more research is recommended [6]. The current Occupational Safety and Health Administration permissible exposure limit is 100 ppm which is based on neurotoxic effects; the current National Institute for Occupational Safety and Health (NIOSH) recommended exposure limit is "the lowest feasible level", which is based on potential carcinogenicity [7].

Numerous studies have examined PCE-exposed workers for adverse health effects (reviewed in [1,2]). Increased risks of scleroderma as well as biliary and liver or brain cancer have been observed as a result of exposure, as have neurological impairments, increased reproductive failure among women including spontaneous abortions, and eccentric sperm morphology and motility among men. Recently, neurobehavioral deficits following PCE exposure have also been reported [8]. Human exposure to PCE sometimes occurs in parallel with other agents and these exposures have been associated with a wide variety of cancers [9-11]. However, it has not been possible to determine whether these human tumors are due to PCE alone, or to other chemicals, or both. Prenatal exposures to PCE have also occurred and have been associated with increases in spontaneous abortions [12,13]. Prenatal exposures to PCE have been assessed in a retrospective cohort study of contaminated drinking water, and no significant effects on birth weight or gestational duration were observed [14]. Similarly, prenatal and early postnatal exposure to PCE-contaminated drinking water were not associated with developmental disorders of attention, learning or behavior identified on the basis of questionnaire responses [15].

Two studies have investigated the cytogenetic effects of PCE in occupationally-exposed humans. Ikeda et al. [16] evaluated chromosomal aberrations and sister chromatid exchanges (SCEs) in lymphocytes from 10 participants. No significant dose-related changes were observed in the frequencies of numerical or structural aberrations, SCEs, or any of several measures of cell growth. Seiji et al. [17] evaluated 27 workers exposed to PCE and did not observe a significant increase in SCE frequencies compared to the unexposed controls.

SCEs and chromosome aberrations are markers of early biologic effects of exposure. However neither endpoint shows persistence beyond a few years following acute exposure. Most types of structural aberrations, including dicentrics and acentric fragments, decline significantly with time after exposure because these aberrations kill cells and as a result their frequencies eventually return to baseline [18-20]. Under conditions of chronic exposure, the induction of SCEs or dicentrics and fragments would be in equilibrium with their loss, possibly reaching a plateau above the baseline levels. However, because of this loss of events over time, neither SCEs nor dicentrics and fragments are accurate biomarkers for quantifying chronic exposure, because detection of the total amount of exposure depends on the accumulation of damage over prolonged periods of time. In contrast, chromosome translocations are compatible with cell survival. For this reason they show much greater persistence through cell division [21,22] and consequently their frequencies are believed to integrate the effect of chronic exposure [23-25]. Thus, while PCE exposure is of concern to human health, investigations of possible cumulative genetic damage resulting from exposure are lacking. For these reasons we sought to evaluate PCE-exposed workers by quantifying translocations, which are not only a persistent biomarker of exposure but also a biomarker of effect because translocations are present in almost all tumors [26]. Here we report the results of a study investigating 18 PCE-exposed female dry cleaners and an unexposed control group comprised of an equal number of female laundry workers who were not exposed to PCE. Cytogenetic analyses for chromosome translocations were performed by whole-chromosome painting in peripheral blood lymphocytes.

## Methods

### Study participants

This study was approved by the NIOSH Human Subjects Review Board. Study design and methods have been described previously [27,28]. Briefly, 18 dry cleaning workers and 18 laundry workers (unexposed controls) were recruited from seven shops in and around southwest Ohio. All participants were women under age 70 who signed an informed consent form and completed an interview which included occupational, smoking, and drinking histories. None of the participants, when asked, reported having had chemotherapy or radiotherapy. Dry cleaners were machine operators or pressers for one year or more (range 1-19, mean 8, standard deviation 5) in shops with third generation machines containing only refrigerated condensers or fourth generation machines with refrigerated condensers and carbon adsorbers [27]. Laundry workers had been in the industry for at least one year and had never been exposed to PCE. Laundry workers were matched by race (Caucasian or African-American), smoking status, and age (+/- 5 years when possible) to already selected dry cleaners. Personal breathing zone samples were collected from dry cleaners on Wednesday of a typical work week. Two or more area samples and personal breathing zone samples were collected from a minimum of two workers in each laundry. Sampling analysis determined concentration of PCE in air according to NIOSH Method 1003 [29].

### Blood collection and air sampling

Venous blood was collected from dry cleaners before work on a Thursday following three consecutive days of PCE exposure, and from launderers on a typical work day. At the same time, pre-shift end-exhaled air was collected and analyzed by NIOSH Method 3704 [30]. The blood specimens were shipped to the PCE-analysis laboratory and refrigerated upon arrival. The average time between blood drawing and analysis was 32 (range 6 to 64) days. All exposure measurements of air levels were performed in accordance with procedures of the NIOSH Analytical Methods Manual [29]. All pre-shift specimens were collected before the dry cleaners entered their dry cleaning shops. Blood was obtained via venipuncture. Blood samples for measuring PCE were collected in gray-top vacutainers that had been previously processed to remove volatile contaminants [31]. These whole blood samples were kept at refrigerator temperature until analysis. Blood PCE was measured by the method of Ashley et al. [31]. Volatile compounds were separated from the blood matrix by purge and trap concentration. Detection, identification, and quantification of PCE was performed by GC/MS. Quantification was achieved by isotope dilution and reference to commercially available standard compounds [31].

### Chromosome painting

For the fluorescent in situ hybridization (FISH) whole chromosome painting analysis, 5 ml blood were obtained in heparinized Vacutainer CPT tubes, and transported at refrigeration temperature to the Lawrence Livermore National Laboratory for arrival within 24 hours of collection. All samples were then immediately set up in culture as described [32] for 52 hours, the last 4 in the presence of 0.1 μg/ml Colcemid. Cells were then swollen with 75 mM KCl as hypotonic, fixed three times in methanol: acetic acid (3:1 v/v), dropped onto clean microscope slides, air dried, then stored in the presence of N_2 _gas and CaSO_4 _desiccant at -20°C until needed for hybridization. Chromosomes 1, 2, and 4 were painted red and chromosomes 3, 5, and 6 were simultaneously painted green, then counterstained with DAPI (blue) as described [32]. An average of 766 (range 751 to 919) whole genome equivalents was scored from each participant. The structural chromosome aberrations scored included translocations, insertions, dicentrics and acentric fragments. Color junctions were also enumerated, and included any chromosome rearrangement that leads to the misunion of chromosome pieces painted in different colors. Most color junctions are the result of translocations, with smaller contributions from insertions, dicentrics and acentric fragments. Complete terminology and specific analytical techniques have been described [32,33].

### Statistical analyses

SAS^® ^(Version 9.1, SAS Institute, Cary, NC, USA) was used to analyze the data. Pearson correlation coefficients were used to determine the unadjusted relationships between the PCE exposure indices (TWA {time-weighted average}, breath, and blood PCE) and the FISH biomarkers (percent of cells with structural chromosome aberrations). Differences in the percent of cells with aberrations (translocations, insertions, dicentrics, acentric fragments, and all exchanges, i.e. color junctions) between exposed and unexposed workers were evaluated with t-tests. We evaluated the percent of cells with each type of aberration, rather than the number of aberrations per cell, because this allowed us to avoid problems with enumerating different types of translocations [22], e.g. differential counting of reciprocal and non-reciprocal exchanges. For the sake of consistency, the other types of aberrations evaluated here are reported in this same manner. Multiple linear regression models were used to determine the adjusted relationships between the natural log blood PCE and each type of aberration. The data were log-transformed to obtain approximate normal distributions. The covariates included in the models were pack years (cumulative smoking index), log cumulative alcohol intake, and age. Positive relationships between exposure and aberration level were considered statistically significant, using one-tailed tests, if p < 0.05.

## Results and Discussion

The demographics of the study participants are shown in Table 1. There were no significant differences between the PCE-exposed dry cleaners and the laundry workers (unexposed controls) for race, current cigarette smoking status, or age. PCE TWA in laundries was below the level of detection (0.023 ppm) for both the personal breathing zone and the area monitors. Post-shift exhaled breath PCE levels in dry cleaners were generally higher than their pre-shift levels. Because blood PCE levels were available both for dry cleaners and laundry workers, and are considered the preferred exposure measure, they were used as the occupational exposure independent variable [27].

**Table 1:** Data Summary.

*Demographics*	Laundry Workers	Dry Cleaners
Facilities	3 ^a^	4
Workers	18 ^a^	18
Race, Caucasian/African American	12/6	13/5
Current smokers	10	10
Age	39 ± 9 ^b^	40 ± 13
***Exposure Indices***		
PCE TWA (ppm) ^c^	<0.02	3.8 ± 5.3
Pre-shift end-exhaled PCE (ppm) ^d^	--	0.45 ± 0.33
Post-shift end-exhaled PCE (ppm) ^d^	--	1.21 ± 0.87
Blood PCE	0.19 ± 0.44 μg/l	74.81 ± 104.27 μg/l ^e^
Pack years^f^	8.44 ± 11.32	13.13 ± 12.0
Lifetime number of drinks^g^	1,106 ± 2,609	6,382 ± 18,144

^a ^Values are number of facilities that contributed participants to this study, workers who are Caucasian or African American, and number of smokers. 20 laundry workers were recruited but only the 18 who donated blood specimens are included here.

^b ^All values are mean ± SD.

^c ^PCE TWA in laundries was below the level of detection (0.023 ppm).

^d ^End-exhaled breath was collected from laundry workers but only one had a PCE level (4.5 ppb) above the limit of detection.

^e ^Mean is significantly different than the corresponding mean for laundry workers (t-test, P < 0.05).

^f ^Packs/day × smoking years.

^g ^Drinks ("cans or glasses of beer, glasses of wine, shots of hard liquor")/week × 52 × drinking years.

**Figure 1:**
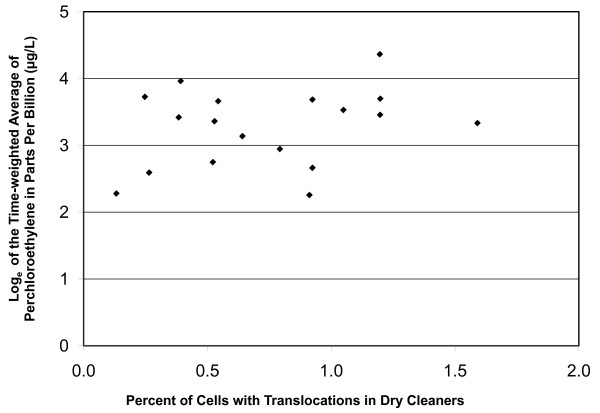
**Translocation frequencies by time-weighted average of PCE in the dry cleaners**. No significant relationship is evident between translocations and blood PCE levels (R^2 ^= 0.26; p = 0.13).

**Figure 2:**
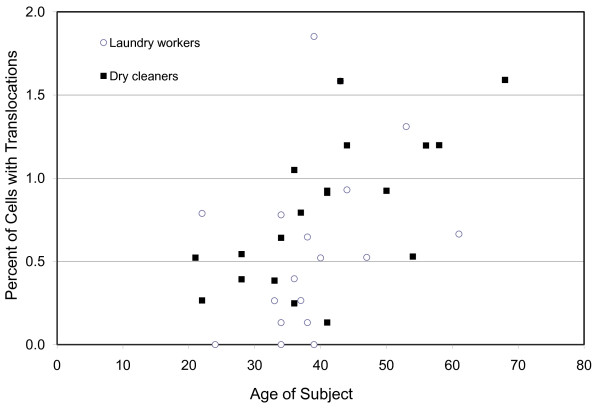
**Translocation frequencies by age in dry cleaners and laundry workers**. A highly significant effect of age is apparent (R^2 ^= 0.52; p = 0.0012).

Regression analyses were performed using age, log of the blood PCE levels, log of the cumulative alcohol consumption, and pack years as the independent variables. A total of five dependent variables were evaluated; these are the percent of cells with chromosome translocations, inversions, dicentrics, fragments, and color junctions. Each dependent variable was regressed again the set of four independent variables. None of the chromosome aberration types showed a significant effect of PCE exposure, or effects due to cigarette smoking or alcohol consumption (data not shown). The absence of an effect of PCE exposure upon translocations is evident in Figure 1. However, as shown in Figure 2, translocations did show a significant effect with age, which is consistent with a recently published report of more than 1900 unexposed participants [34]. Color junctions also showed a significant effect with age but this is likely to be due to the inclusion of translocations in this endpoint as the frequencies of dicentrics, fragments, and insertions showed no age effect.

**Figure 3:**
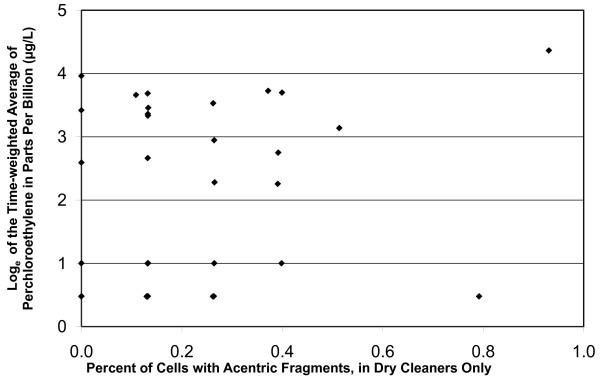
**Frequencies of acentric fragments by time-weighted average of PCE in the dry cleaners**. None of the laundry workers had acentric fragments. The frequencies of acentric fragments and blood PCE levels are correlated (R^2 ^= 0.49; p = 0.0026).

Correlation analyses were also performed among the variables. PCE blood level, TWA of the blood PCE levels, and exhaled breath were highly correlated [27]. No correlation was observed between the TWA of PCE and the percent of cells with chromosome translocations, inversions, dicentrics, and color junctions. However, PCE levels were significantly correlated with acentric fragments (p = 0.0026, Figure 3). In addition, the dry cleaners had higher percentages of cells with translocations, insertions, color junctions, dicentrics and acentric fragments than did the laundry workers but none of these increases were statistically significant (Table 2).

**Table 2:** Cytogenetic comparisons of 18 dry-cleaning workers exposed to tetrachloroethylene and 18 unexposed laundry workers.

Variable	PCE exposed?	Percent	Confidence Interval	Pooled t-test	Pr > |t|*
% cells with translocations	no	0.599	(0.329 - 0.870)	-0.93	0.18
	
	yes	0.747	(0.546 - 0.948)		

					

% cells with insertions	no	0.0073	(-0.008 - 0.0229)	-1.18	0.12
	
	yes	0.0291	(-0.007 - 0.0648)		

					

% cells with color junctions	no	0.621	(0.338 - 0.905)	-1.13	0.13
	
	yes	0.811	(0.597 - 1.025)		

					

% cells with dicentrics	no	0.0365	(0.0064 - 0.0667)	-1.99	0.027
	
	yes	0.0873	(0.0426 - 0.132)		

					

% cells with acentric fragments	no	0.161	(0.0641 - 0.258)	-1.30	0.10
	
	yes	0.253	(0.140 - 0.367)		

* Probability of a positive relationship between exposure and level of aberrations (one-tailed test).

The dry cleaning plants where the exposed workers were employed had low ambient levels of PCE, and only one of the exposed workers was a full-time machine operator. Since operators have much higher exposures than do other employees, and since many shops have higher ambient PCE levels, this does not rule out a PCE-exposure effect among more heavily exposed workers.

## Conclusions

In this population there does not appear to be a strong effect of PCE exposure on chromosome damage, even for translocations that measure accumulated exposure. However, the dry cleaners did have non-significant increases in translocations, insertions, fragments, dicentrics and junctions compared to laundry workers. Further work on more heavily exposed participants and with a larger sample size will be needed to determine whether PCE exposure induces significant levels of cytogenetic damage.

## List of Abbreviations

PCE: tetrachloroethylene, perchloroethylene; NIOSH: National Institute for Occupational Safety and Health; SCEs: sister chromatid exchanges; FISH: fluorescence in situ hybridization; TWA: time-weighted average

## Competing interests

The authors declare that they have no competing interests.

## Authors' contributions

JDT oversaw the cytogenetic portion of this work and wrote the first draft of the paper. KJS performed the tissue culturing and the cytogenetics work. LTM contributed to the original protocol, oversaw specimen collection, and conducted breath analyses. AMR contributed to the original protocol, participated in the field study, and did statistical analyses. CLF and MAB contributed to the original protocol and participated in the field study. All authors have read and approved the final manuscript.
